# Interaction of Plants and Endophytic Microorganisms: Molecular Aspects, Biological Functions, Community Composition, and Practical Applications

**DOI:** 10.3390/plants12040714

**Published:** 2023-02-06

**Authors:** Olga A. Aleynova, Konstantin V. Kiselev

**Affiliations:** Laboratory of Biotechnology, Federal Scientific Center of the East Asia Terrestrial Biodiversity, Far Eastern Branch of the Russian Academy of Sciences, 690022 Vladivostok, Russia

Endophytes are microorganisms that live asymptomatically inside plant tissues. Plant endophytes are mainly represented by bacteria and fungi, while archaebacteria, algae, protozoa, viruses and nematodes are rarely found to be living as endophytes. Endophytes are distributed in all plant organs such as roots, stems, leaves, seeds and fruits. Endophytes have been found in every plant species that has been studied. Nearly 300,000 plant species that exist on earth are thought to be a host to one or more endophytes.

To become endophytes, microorganisms should colonize the plant endosphere after first colonizing the rhizosphere. This process of colonization is achieved using the specific properties of microorganisms involving motility, attachment, plant–polymer degradation and the evasion of plant defenses. The diversity of endophytic community formed by various microorganisms and depends on plant- and environment-specific factors. The consortium of endophytes can be represented by the different types of endophytes in the same plant species. Geographic location, season, climate and type of plant tissue are among the factors that affect species composition and the frequency of endophyte colonization.

The interactions between endophytes and their plant hosts are diverse. Plants provide protection to endophytes, and most endophytic microorganisms have no effect on plants. Some endophytes can act as pathogens, while others exhibit beneficial properties for plants. Endophytes are capable of producing useful metabolites such as phosphorous, iron (siderophores), and nitrogen from the environment or generating growth-regulating phytohormones such as auxins, citokinins, gibberellic acid and ethylene (ACC deaminase). These endophytes increase plant nutrient uptake and induce plant resistance to pathogens, osmotic stress, heavy metals, xenobiotic contaminants and other forms of abiotic stress. Moreover, endophytes can improve plant health by targeting pests and pathogens with antibiotics, hydrolytic enzymes, and nutrient limitation and by priming plant defenses. In fact, a plant without the associated endophytes would be less fit to deal with phytopathogens and more susceptible to the stress conditions. However, there is still insufficient information about the interaction of plants and endophytes.

In recent years, plant endophytes have attracted more attention in terms of their diversity and application for improving plant properties or plant disease protection. The broad host range of endophytes makes them a powerful tool in agricultural biotechnology. Some endophytes can be used as bioinoculants in developing a safe and sustainable agriculture system. The beneficial substances produced by endophytes can serve as sources of new natural products for medicinal, agricultural and industrial purposes. Therefore, endophytes have great potential to be used as biofertilizers and biopesticides in developing a sustainable, safe and effective agriculture system.

This Special Issue presents studies that highlight different aspects of the plant–endophyte interaction. Most of the articles report on the biodiversity of endophytic bacteria and fungi of various plant species [1,2,3,4,5,6,7,8]. The review article elaborates interesting data on the biodiversity of bacterial, fungal, archaeal and viral endophytes in ginseng and data on ginseng interactions with bacterial and fungal endophytes [1]. Another interesting work was included as a study of rhizosphere microbiota of areca palm (*Areca catechu*) at different stages of plant development [2]. Additionally, changes in the diversity and composition of the community of root endophytic fungi associated with *Aristolochia chilensis* along the aridity gradient in the Atacama Desert have been studied [3]. A study of the diversity of fungi involved in the damage of Japanese quince is presented in this Special Issue [4]. In addition, this Special Issue presents three publications on the biodiversity of endophytes in grapes [5,6,7], including an analysis of the biodiversity of bacterial endophytes in the wild grape *Vitis amurensis* growing in Russia [5], the rhizosphere microflora of nine *Vitis vinifera* grape cultivars, including Zinfandel, Cabernet Sauvignon, Syrah, Merlot, Gem, Pinot noir, Riesling, Longan, Chardonnay in northern China [6], and relationships among grapevine *V. vinifera* cultivars (Furmint, Kadarka, and Syrah) chemical and physiological parameters in relation to leaf and berry mycobiome composition [7].

One publication developed the molecular basis for plant–bacteria interactions using dual RNA-seq analyses [8]. In this study, dual RNA-seq analyses were performed to provide insights into the early-stage interactions between barley seedlings and three novel bacterial strains (two *Paenibacillus* sp. strains and one *Erwinia gerundensis* strain) isolated from the perennial ryegrass seed microbiome [8].

Moreover, two publications are concerned with the study of the effect of endophytes on plant secondary metabolism [9,10]. These studies described the influence of the grapevine bacterial and fungal endophytes on stilbene production in grape cells [9], the synthesis of cytotoxic macrolide maytansine by endophytes inside their plant host [10], and the impact of maytansine production on plant secondary metabolites [10].

Additionally, this Special Issue contains a number of papers aimed at studying the activity of endophytic bacteria and fungi in comparison to known plant pathogens [11,12,13]. These studies described the nematocidal activity of the endophyte *Serratia ureilytica* against *Nacobbus aberrans* [11], antagonistic activity of fungal strains (in particular, *Talaromyces trachyspermus*) against *Fusarium* crown Rot [12] and fungicidal activity of volatile organic compounds isolated by two beneficial endophytic *Pseudomonas* strains from olive roots [13].

In addition, five papers are concerned with studying the relationship between the quality of the soil and the substances introduced into it via the endophytic biodiversity of plants [14,15,16,17,18]. The rhizosphere and endophytic microbiome of the bamboo plant were evaluated in response to the prolonged application of heavy organic fertilizers [14]. It was characterized root-associated microbiomes of rice plants during co-planted barnyard grass stress and a comparison with the microbiomes of unplanted soil [15]. The results of a comprehensive study on the prevention and control of clubroot disease (caused by *Plasmodiophora brassicae*) in Chinese cabbage through crop rotation with marigold [16] were described for the first time. Additionally, we habe presented results from a temporal variability in late blight pathogen diversity, virulence and fungicide resistance in potato breeding fields [17]. An interesting study was also conducted which demonstrated that *Variovorax* can be used as a biofertilizer to improve the adaptation of legumes to degraded soils in soil recovery programs [18].

Understanding plant–endophyte interactions will require additional research. In particular, researchers must study the physiological and molecular aspects of such interactions, as well as their biological functions and practical applications. Many issues still need to be explored in this respect. This Special Issue represents a collection of some most intensively studied topics for the interaction of plants with endophytic microorganisms.

## Data Availability

https://www.mdpi.com/journal/plants/special_issues/plants_endophytic_microorganisms.
